# Time Course of Peripheral Leukocytosis and Clinical Outcomes After Aneurysmal Subarachnoid Hemorrhage

**DOI:** 10.3389/fneur.2021.694996

**Published:** 2021-07-26

**Authors:** Aaron M. Gusdon, Jude P. J. Savarraj, Eyad Shihabeddin, Atzhiry Paz, Andres Assing, Sang-Bae Ko, Louise D. McCullough, Huimahn Alex Choi

**Affiliations:** ^1^Department of Neurosurgery, McGovern Medical School, University of Texas Health Science Center, Houston, TX, United States; ^2^Department of Neurology, Seoul National University College of Medicine, Seoul, South Korea; ^3^Department of Neurology, McGovern Medical School, University of Texas Health Science Center, Houston, TX, United States

**Keywords:** monocytes, neutrophil-to-lymphocyte ratio, subarachnoid hemorrhage, neuroinflammation, delayed cerebral ischemia

## Abstract

**Objective:** Systemic inflammation after subarachnoid hemorrhage (SAH) is implicated in delayed cerebral ischemia (DCI) and adverse clinical outcomes. We hypothesize that early changes in peripheral leukocytes will be associated with outcomes after SAH.

**Methods:** SAH patients admitted between January 2009 and December 2016 were enrolled into a prospective observational study and were assessed for Hunt Hess Scale (HHS) at admission, DCI, and modified Ranked Scale (mRS) at discharge. Total white blood cell (WBC) counts and each component of the differential cell count were determined on the day of admission (day 0) to 8 days after bleed (day 8). Global cerebral edema (GCE) was assessed on admission CT, and presence of any infection was determined. Statistical tests included student's *t*-test, Chi-square test, and multivariate logistic regression (MLR) models.

**Results:** A total of 451 subjects were analyzed. Total WBCs and neutrophils decreased initially reaching a minimum at day 4–5 after SAH. Monocyte count increased gradually after SAH and peaked between day 6–8, while basophils and lymphocytes decreased initially from day 0 to 1 and steadily increased thereafter. Neutrophil to lymphocyte ratio (NLR) reached a peak on day 1 and decreased thereafter. WBCs, neutrophils, monocytes, and NLR were higher in patients with DCI and poor functional outcomes. WBCs, neutrophils, and NLR were higher in subjects who developed infections. In MLR models, neutrophils and monocytes were associated with DCI and worse functional outcomes, while NLR was only associated with worse functional outcomes. Occurrence of infection was associated with poor outcome. Neutrophils and NLR were associated with infection, while monocytes were not. Monocytes were higher in males, and ROC curve analysis revealed improved ability of monocytes to predict DCI and poor functional outcomes in male subjects.

**Conclusions:** Monocytosis was associated with DCI and poor functional outcomes after SAH. The association between neutrophils and NLR and infection may impact outcomes. Early elevation in monocytes had an improved ability to predict DCI and poor functional outcomes in males, which was independent of the occurrence of infection.

## Introduction

Aneurysmal subarachnoid hemorrhage (SAH) results in significant morbidity (1). Delayed cerebral ischemia (DCI) is a late complication occurring typically 4–14 days after onset in one-third of SAH caused by a combination of angiographic vasospasm, arterial constriction and thrombosis, and cortical spreading ischemia (2, 3) and is a major contributor to clinical outcomes (4, 5). Early brain injury (EBI) occurring within 72 h after aneurysmal rupture has been shown to be predictive of clinical outcomes (6). Global cerebral edema (GCE) quantifies the effacement of hemispheric sulci, and is a radiographic marker of early brain injury (7).

Uncontrolled inflammation occurs during EBI due to the reaction to extravascular blood (8), impaired cerebral autoregulation (9), release of products from injured brain tissue, and ischemia-reperfusion injury (7). Pro-inflammatory cytokines such as interleukin-1β (IL-1β) triggers the release of astrocyte derived extracellular vesicles that enter the peripheral circulation and promote the transmigration of leukocytes to the central nervous system (CNS) (10). The subsequent robust systemic inflammatory response occurring after SAH peaks at 24–48 h and contributes to delayed neurological deterioration (11, 12).

Peripheral leukocytes have been shown to migrate to the cerebrospinal fluid and brain after SAH (13) with activated neutrophils damaging brain micro-vessels (14). Preclinical models have shown that neutrophil and monocyte levels peaked by day 5 post SAH and that the leukocytes found in the brain originated from systemic blood circulation (5, 15). Early elevation in total peripheral leukocytes has been linked with the occurrence of DCI and poor functional outcomes (16, 17). However, leukocyte subtypes likely play distinct roles. After intracerebral hemorrhage (ICH), increased peripheral monocyte counts as well as neutrophil to lymphocyte ratios (NLR) are associated with worse outcomes (18–21). Similarly, after SAH, numerous studies have demonstrated that increased NLR is associated with worse outcomes (22–26). Peripheral monocytosis has also been linked to development of DCI (27). However, the biological mechanism underpinning the associations between leukocytes and outcomes and the link between inflammatory cells and EBI remain poorly understood.

The objective of this study is to investigate trends in leukocyte counts after SAH and the associations between leukocytes and outcomes. Total WBC count, monocytes, leukocytes, neutrophils, and basophils were collected at admission and each day for the next 8 days. We hypothesized that increased peripheral inflammatory cells will be associated with poor clinical outcomes and DCI.

## Materials and Methods

### Study Population, Inclusion, and Exclusion Criteria

This is a retrospective, observational, single center study of SAH patients admitted between January 2009 and December 2016 to the Neuroscience Intensive Care Unit at Memorial Hermann Hospital, University of Texas (UT) Health Science center at Houston. Ethical approval was obtained from the UT Health Science Center institutional review board (17-0776). Inclusion criteria for the study were: presence of SAH on initial CT or presence of xanthochromia in cerebrospinal fluid, age above 18 years, and admission to hospital within 72 h of bleed ictus. Exclusion criteria for the study included: SAH associated with trauma, arteriovenous malformation, or mycotic aneurysms; presence of auto-immune diseases; and conditions that affect inflammation such as pregnancy or a history of malignancy. Subjects with perimesencephalic SAH were excluded due to the low likelihood of aneurysmal etiology and low risk of aneurysmal etiology and development of DCI (28).

### Demographic, Clinical, and Radiographic Data

Subjects' demographics and clinical information were abstracted from the electronic medical record. The Hunt Hess Scale (HHS) score was used to quantify clinical severity on admission (29). Patients were dichotomized into good grade group (HH ≤ 3) and poor grade groups (HH ≥4). All patients were monitored for DCI. DCI is a dichotomous score (either 0 or 1) defined as “the occurrence of focal neurological impairment or a decrease of at least 2 points on the Glasgow Coma Scale that lasts for at least 1 h, is not apparent immediately after aneurysm occlusion, and cannot be attributed to other causes by means of clinical assessment, CT or MRI scanning of the brain, and appropriate laboratory studies” (30). The 0–6 modified Rankin score (mRS) was used to quantify functional outcome of patient at the time of discharge. Patients were dichotomized into good (mRS ≤ 3) and poor (mRS ≥4) clinical outcomes. Infection was defined as the presence of ventriculitis, urinary tract infection (UTI), or pneumonia.

### Radiographic Markers of Injury

Initial computed tomography (CT) scans at the time of SAH diagnosis were graded for parameters including global cerebral edema (GCE), intraventricular hemorrhage (IVH), and modified Fisher Scale (mFS). GCE is an important radiographic marker of EBI which is also a risk factor for DCI (7). It is a qualitative and dichotomous score (either 0 or 1) based on the radiographic presence of [1] complete or near-complete effacement of hemispheric sulci and basal cisterns or [2] bilateral and extensive disruption of the hemispheric gray-white matter junction at the level of the central semi vale, which is due to either blurring or diffuse peripheral “fingerlike” extensions of the normal demarcation between gray and white matter (7). IVH is a qualitative and dichotomous radiographic score (either 0 or 1) defined as the presence or bleeding inside or around the ventricles in the brain that normally contain cerebral spinal fluid. GCE, IVH, and mRS were determined by at least two independent physicians, with at least one being an attending neurointensivist. All scores were adjudicated at a weekly research meeting.

### Leukocytes Subtypes and Time Course

Complete blood counts (CBC) with differential were collected as a part of routine patient care and management. Differentials included total white blood cells (WBC), neutrophils, monocytes, lymphocytes, eosinophils, and basophils. Daily values for each cell type were abstracted from day of admission (day 0) to day 8. The early (within 48 h) period most likely corresponds with the occurrence of EBI after SAH (15–17). Days 3–5 reflect the time period immediately preceding the occurrence of DCI. The 6–8-day period represents the peak period of DCI. Given that leukocyte counts at different time points after injury may play distinct roles, we created a variety models using a variety of different time points. Models were created for cells counts on each individual day. Models were also created using median cell counts over days 1–5, as this timeframe was thought to precede development of DCI in most patients.

### Statistical Analysis

Student's *t*-tests were performed on continuous variables while χ^2^ tests were performed on categorical variables. *p* < 0.05 were considered to be significant. Univariate models were created to assess the association between each cell type over days 0–8. Given that 54 comparisons were made, a Bonferroni correction was used to set significance at a *P*-value of < 9.3 × 10^−4^ for univariate models. Multivariable logistic regression (MLR) models were used to determine independent associations after controlling for variables found to be significant in univariate models. Models presented herein reflect median cell counts over days 1–5, unless otherwise specified. Receiver operating characteristic (ROC) curves were calculated with an area under the curve (AUC) calculated for each. RStudio (v1.2.5033) was used for all statistical analysis.

## Results

### Demographics and Outcomes

A total of 451 patients met the inclusion criteria and were included in this study. Demographic characteristics and clinical outcomes of subjects are shown in Table 1. The average age was 54 (IQR 45, 63), 295 (65%) were female, 304 (67%) had hypertension, 240 (53%) presented with IVH, and 95 (21%) had HH ≥4 at admission. Aneurysms were treated by surgical clipping in 139 (31%) and by coiling in 222 (49%). No surgical treatment for aneurysm was undertaken in 32 (7%), and no aneurysm was found in 58 (13%). Subjects who developed DCI had higher mFS scores [mFS ≥3: 232 (64%) vs. 75 (85%), *P* < 0.001], higher proportion of IVH [173 (48%) vs. 67 (76%), *P* < 0.001], higher frequency of aneurysm clipping [101 (28%) vs. 38 (43%), *P* = 0.041], worse outcomes [mRS ≥4: 97 (27%) vs. 49 (56%), *P* < 0.001], and longer hospital length of stay [12 days (IQR 9, 16) vs. 19 days (14, 24), *P* < 0.001]. Subjects with poor outcomes (mRS ≥4) were older [age 51 (IQR 42, 59) vs. 61 (31, 32), *P* < 0.001], had a higher proportion of hypertension [197 (65%) vs. 107 (73%), *P* = 0.004], had higher HH grade [HH 4,5: 21 (6.9%) vs. 74 (51%), *P* < 0.001], had higher mFS scores [mFS ≥3: 186 (61%) vs. 121 (83%), *P* < 0.001], had a higher proportion of IVH [133 (44%) vs. 107 (73%), *P* < 0.001], had a higher incidence of DCI [39 (13%) vs. 49 (34%), *P* < 0.001], and had a higher hospital length of stay [12 days (IQR 9, 16) vs. 16 days (IQR 8, 23), *P* < 0.001].

**Table 1:** Demographics and clinical outcomes.

	**Total**	**DCI (-)**	**DCI (+)**	***P***	**mRS (0-3)**	**mRS (4-6)**	***P***
**N**	451	363	88		305	146	
**Demographics**							
Age	54 (45, 63)*	54 (44, 64)	54 (46, 62)	0.903	51 (42, 59)	61 (51, 71)	** <0.001**
Gender (*F*)	295 (65)	235	60	0.436	197 (65)	98 (67)	0.441
Hypertension	304 (67)^†^	248 (68)	56 (64)	0.461	197 (65)	107 (73)	**0.004**
HHS (4,5)	95 (21)	71 (20)	24 (27)	0.112	21 (6.9)	74 (51)	** <0.001**
mFS (3,4)	406 (90)	232 (64)	75 (85)	** <0.001**	186 (61)	121 (83)	** <0.001**
IVH	240 (53)	173 (48)	67 (76)	** <0.001**	133 (44)	107 (73)	** <0.001**
Aneurysm treatment:				**2.83** **×** **10**^**−6**^			**8.00** **×** **10**^**−9**^
Clipped	139 (31)	101 (28)	38 (43)	**0.041**	99 (32)	40 (27)	0.999
Coiled	222 (49)	172 (47)	50 (57)	0.898	146 (48)	76 (52)	0.999
No treatment	32 (7)	32 (9)	0 (0)	**0.031**	8 (3)	24 (16)	**1.00** **×** **10**^**−6**^
No aneurysm	58 (13)	58 (16)	0 (0)	**0.0005**	52 (17)	6 (4)	**9.81** **×** **10**^**−4**^
**Outcomes**							
DCI	88 (20)				39 (13)	49 (34)	** <0.001**
mRS (4-6)	146 (32)	97 (27)	49 (56)	** <0.001**			
Hospital LOS	13 (9, 18)	12 (9, 16)	19 (14, 24)	** <0.001**	12 (9,16)	16 (8, 23)	**0.001**

Data are presented as either ^*^median (interquartile range) or^†^N (percent). HHS, Hunt Hess Scale; mFS, modified Fisher Scale; IVH, intraventricular hemorrhage; DCI, delayed cerebral ischemia; mRS, modified Rankin Scale; LOS, length of stay. P-values that are statistically significant are in bold.

### Leukocyte Trends

Overall trends in leukocytes are shown in Supplementary Figure 1. WBC count was highest on day 0 after SAH, decreased until day 5, and then increased again. This same trend occurred for neutrophils. Monocytes increased gradually after SAH, peaking at day 8. After an initial decrease from day 0 to day 1, basophil count increased gradually until peaking at day 7. Lymphocytes also decreased from day 0 to day 1, followed by an increase reaching a peak at day 7. NLR was highest at day 1 after SAH, and then decreased to reach a minimum at day 6 after SAH, subsequently increasing again thereafter.

### Associations With Disease Severity and Outcomes

Leukocytes stratified by HHS are shown in Supplementary Figure 2. In subjects with higher HHS (4, 5) on admission, WBC count was significantly higher on all days except day 5. Neutrophils were significantly higher in subjects with higher HHS across all days. Monocytes were higher in subjects with higher HHS on days 0, 1, and 2. There were no significant differences in basophils. Lymphocytes higher in subjects with higher HHS on day 0 and were lower on days 3, 4, 5, and 6. NLR was higher in subjects with higher HHS on all days except for day 0.

**Figure 1:**
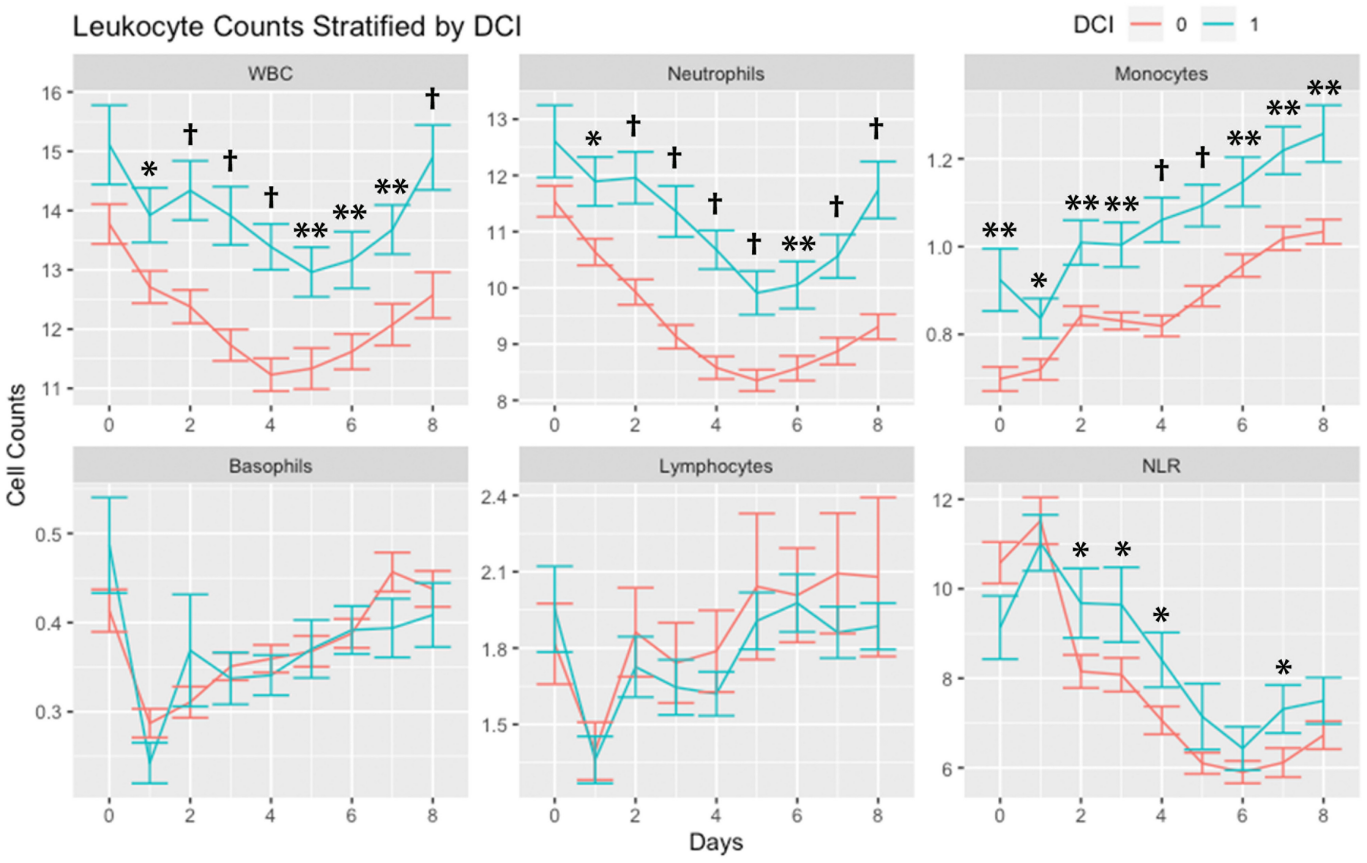
Leukocyte counts stratified by occurrence of delayed cerebral ischemia (DCI). Leukocytes counts (mean and standard deviation) are shown from day of SAH (day 0) through day 8. Cells are in units of 1,000 per mm^3^. *P < 0.05, **P < 0.01,^†^*P* < 0.001. Abbreviations: delayed cerebral ischemia (DCI), subarachnoid hemorrhage (SAH), white blood cells (WBC), neutrophil-to-lymphocyte ratio (NLR).

Leukocytes stratified by occurrence of DCI are shown in Figure 1. WBC and neutrophil counts were higher in subjects with DCI across all days with the exception of day 0. Monocytes were higher in subjects with DCI across all days. There were no differences in basophils and lymphocytes among subjects with and without DCI. NLR was higher in subjects with DCI on days 2, 3, 4, and 7.

**Figure 2:**
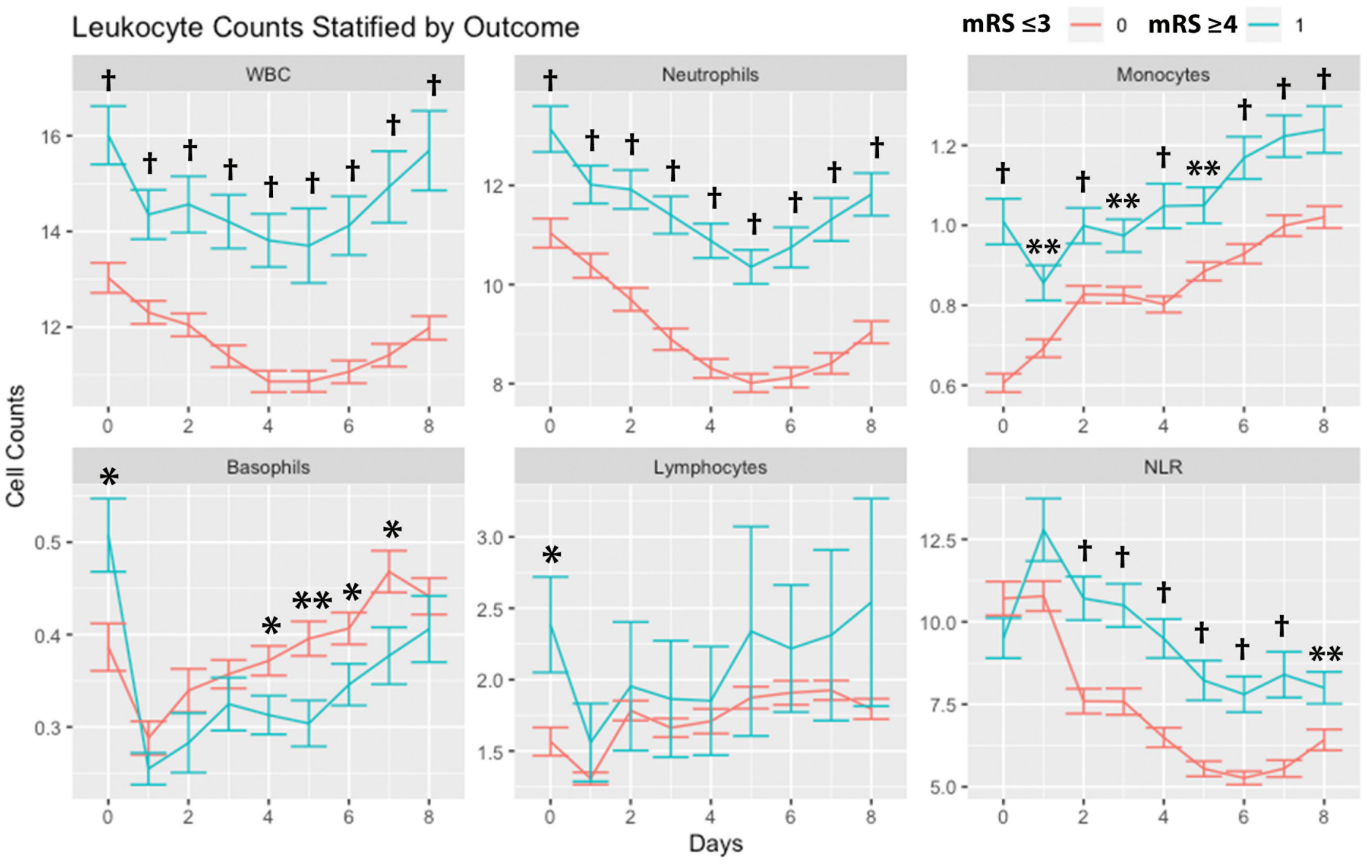
Leukocyte counts stratified by outcomes. Leukocyte counts (mean and standard deviation) are shown from day of SAH (day 0) through day 8. Outcome is dichotomized according to mRS good (≤ 3) or poor (≥4). *P < 0.05, **P < 0.01,^†^*P* < 0.001. Cells are in units of 1,000 per mm^3^. mRS, modified Rankin Scale; SAH, subarachnoid hemorrhage; WBC, white blood cells; NLR, neutrophil-to-lymphocyte ratio.

Leukocytes stratified by outcomes (mRS 0-3 vs. 4-6) are shown in Figure 2. WBCs, neutrophils, and monocytes were higher in subjects with poor mRS outcomes across all days. Basophils were higher on day 0 in patients with poor mRS outcomes, while on days 4, 5, 6, and 7 basophils were higher in subjects with good mRS outcomes. Lymphocytes were higher on day 0 in patients with poor mRS outcomes. NLR was higher in subjects with poor mRS outcomes on all days except days 0 and 1.

### Association With Infection

**Figure 3:**
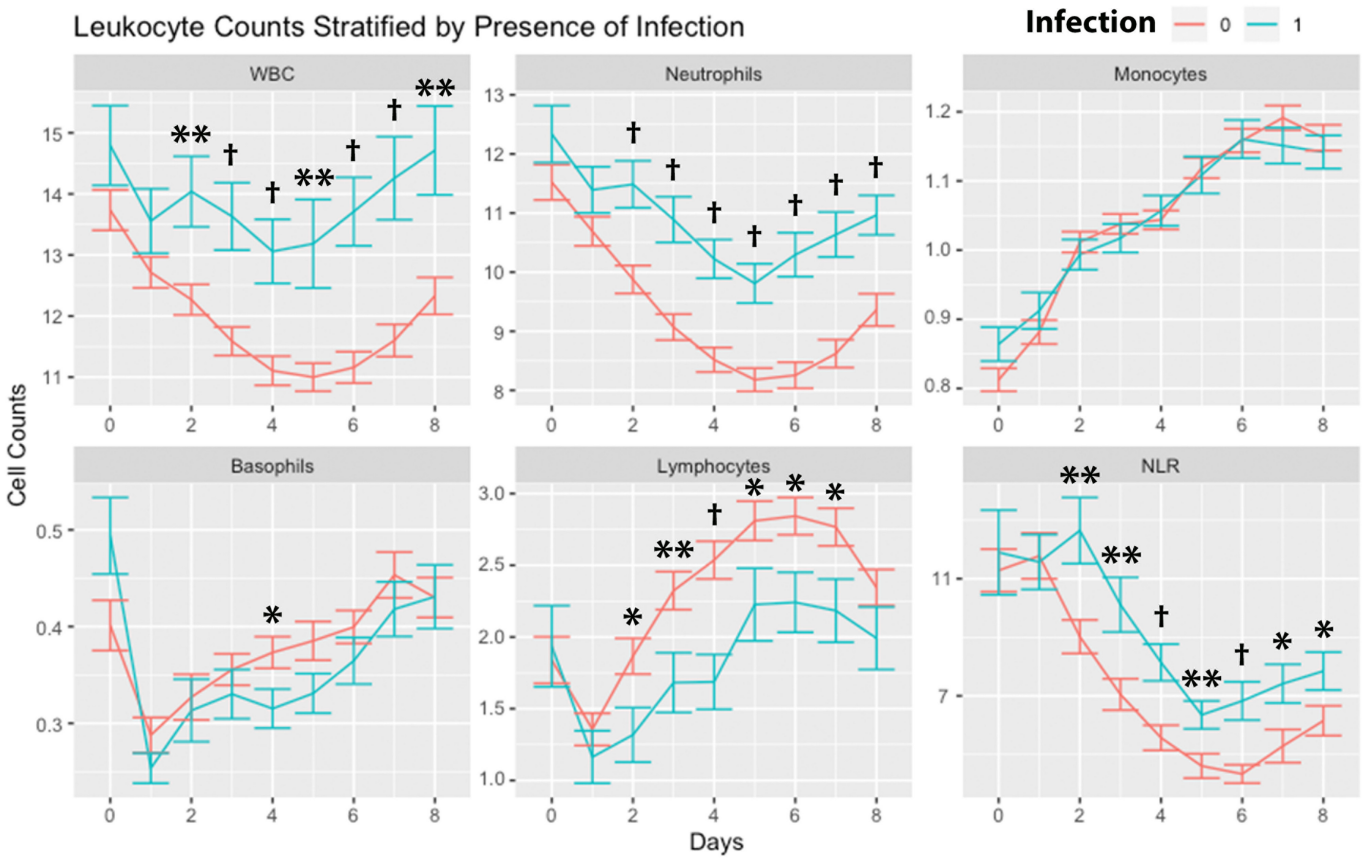
Leukocyte counts stratified by infection. Leukocyte counts (mean and standard deviation) are shown from the day of SAH (day 0) through day 8. Counts are stratified by the occurrence of any infection within days 0–8. *P < 0.05, **P < 0.01,^†^*P* < 0.001. Cells are in units of 1,000 per mm^3^. SAH, subarachnoid hemorrhage; WBC, white blood cells; NLR, neutrophil-to-lymphocyte ratio.

Leukocytes were stratified by the presence of any infection (Figure 3). WBCs and neutrophils were higher in subjects with any infection on day 2 through day 8. There were no significant differences in monocytes. Basophils were higher in subjects without infection on day 4. Lymphocytes were higher in subjects without infection on days 2, 3, 4, 5, 6, and 7. NLR was higher in subjects with infection on days 2 through day 8.

### Sex Differences

**Figure 4:**
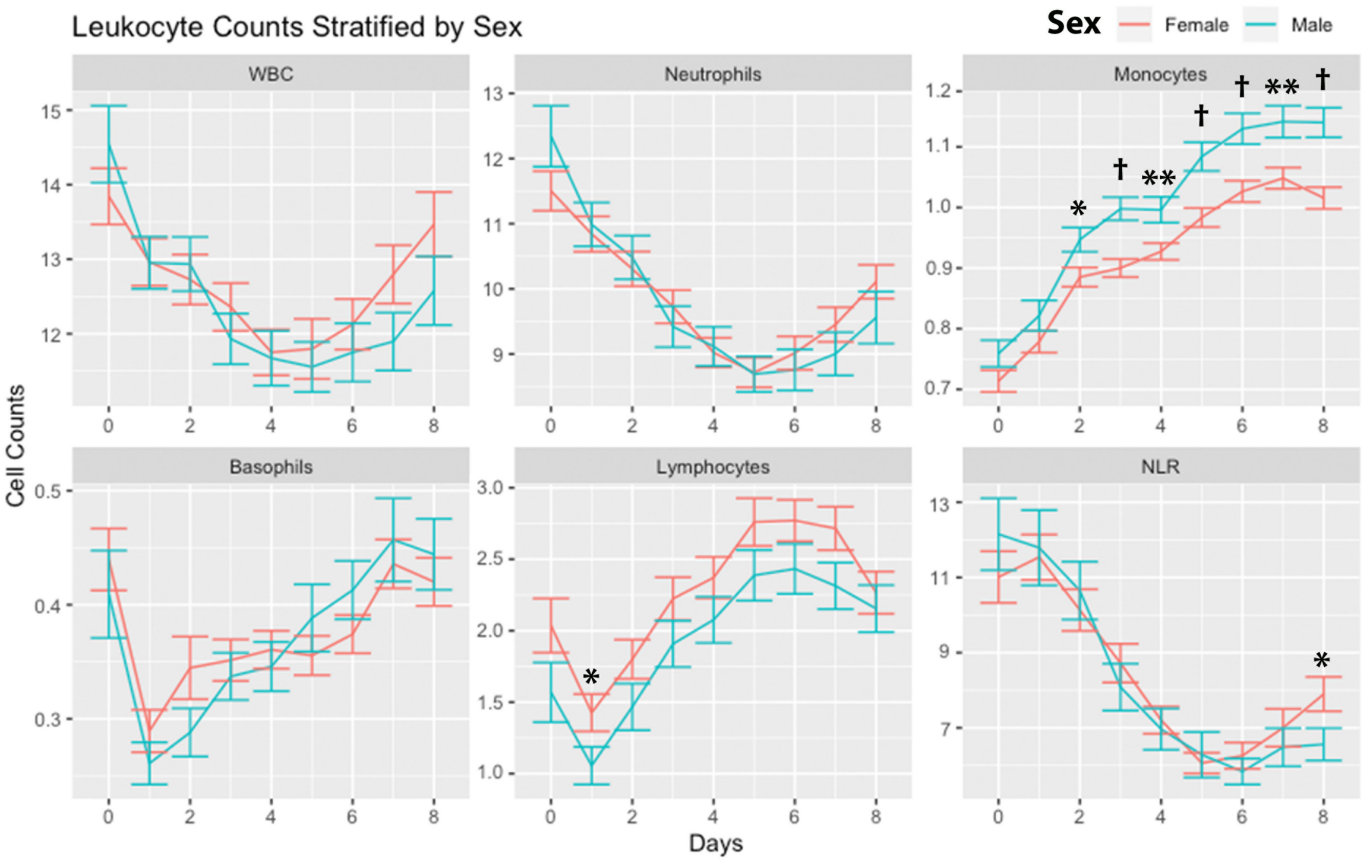
Leukocyte counts stratified by sex. Leukocyte counts (mean and standard deviation) are shown from the day of SAH (day 0) through day 8. Subjects are divided into males and females. *P < 0.05, **P < 0.01,^†^*P* < 0.001. Cells are in units of 1,000 per mm^3^. SAH, subarachnoid hemorrhage; WBC, white blood cells; NLR, neutrophil-to-lymphocyte ratio.

Leukocytes were stratified according to sex (Figure 4). There were no significant differences in WBCs and neutrophils comparing males and females. Monocytes were higher in males on days 2 through day 8. There were no significant differences in basophils. Lymphocytes were higher in females on day 1. NLR was higher in females on day 8.

### Outcome Models

Univariate models were created for each cell count on each day to assess associations with DCI (Supplementary Table 1) and mRS (Supplementary Table 2). Increased WBCs were associated with DCI on days 3 and 4 and neutrophils were associated with DCI on days 2, 3, 4, and 5. Increased monocytes were associated with DCI on all days except day 1. No associations were found for DCI among basophils, lymphocytes, or NLR on any day. Increased WBCs, neutrophils, and monocytes were associated with poor outcomes (mRS 4-6) on all days, while increased NLR was associated with poor outcomes on all days except days 0, 1, and 8. No associations with outcomes were found for basophils and lymphocytes.

MLR models were constructed to assess the associations between cell counts and outcomes. Models were created taking the median cell counts across days 1–5. After correction for covariates, neutrophils and monocytes were associated with DCI, while NLR was not (Table 2). IVH was associated with DCI in the model including monocytes and NLR. Occurrence of infection was associated with DCI only the NLR model. GCE was associated with DCI only in the model containing monocytes. Aneurysm treatment by surgical clipping was associated with DCI in the monocyte and NLR models. Neutrophils, monocytes, and NLR were associated with poor outcomes (mRS 4-6) (Table 3). Increased age, HHS, and presence of any infection were also associated with worse outcomes in each model. IVH was associated with poor outcomes in each model. GCE was associated with poor outcomes in the monocyte and NLR models. Aneurysmal clipping was not associated with function outcome in any model. No significant effect of sex was seen in models for DCI or functional outcomes (Tables 2, 3).

**Table 2:** Associations between leukocytes and DCI.

	Neutrophils^*^	**Monocytes**	**NLR**
Unadjusted	0.19 [0.15, 0.24 (**6.05** **×** **10**^**−6**^)]^#^	2.00 [1.52, 2.48 (**2.71** **×** **10**^**−5**^)]	0.05 [0.03, 0.07 (**0.027**)]
Adjusted	0.18 [0.13, 0.23 (**3.45** **×** **10**^**−4**^)]	1.50 [1.03, 1.97 (**0.0144**)]	0.03 [−0.003, 0.07 (0.358)]
**Covariates**
Age	0.004 [−0.008, 0.01 (0.692)]	0.009 [−0.003, 0.02 (0.444)]	0.007 [−0.004, 0.02 (0.553)]
HHS	0.41 [0.05, 0.76 (0.553)]	−0.06 [−0.45, 0.32 (0.867)]	0.23 [−0.13, 0.50 (0.517)]
mFS	−0.44 [−0.92, 0.05 (0.303)]	0.26 [−0.24, 0.74 (0.406)]	0.36 [−0.03, 0.75 (0.274)]
IVH	0.95 [0.60, 1.30 (**0.008**)]	1.05 [0.66, 1.35 (**0.009**)]	1.04 [0.68, 1.35 (**0.002**)]
GCE	0.68 [0.29, 1.10 (0.094)]	0.88 [0.42, 1.23 (**0.047**)]	0.71 [0.32, 1.10 (0.061)]
Sex (Male)	0.05 [−0.29, 0.35 (0.866]	−0.04 [−0.36, 0.23 (0.913)]	0.008 [−0.30, 0.31 (0.979)]
Any infection	0.52 [0.21, 0.82 (0.092)]	0.31 [−0.10, 1.12 (0.088)]	0.61 [0.32, 0.91 (**0.036**)]
Aneurysm clipping	0.24 [−0.03, 0.51 (0.106)]	0.74 [0.47, 1.01 (**0.007**)]	0.64 [0.37, 0.90 (**0.061**)]

* *Models based on median cell count for days 1–5*.

# *Data are presented as β coefficient [95% confidence interval (P value)]*.

*HHS, Hunt Hess Scale; mFS, modified Fisher Scale; IVH, intraventricular hemorrhage; GCE, global cerebral edema. P-values that are statistically significant are in bold*.

**Table 3:** Associations between leukocytes and outcomes (mRS 4-6).

	Neutrophil Count^*^	**Monocyte Count**	**NLR**
Unadjusted	0.23 [0.18, 0.27 (**4.31** **×** **10**^**−7**^)]^#^	1.88 [1.42, 2.34 (**3.87** **×** **10**^**−5**^)]	0.10 [0.07, 012 (**1.56** **×** **10**^**−5**^)]
Adjusted	0.23 [0.17, 0.28 (**4.98** **×** **10**^**−5**^)]	1.36 [0.83, 1.91 (**0.009**)]	0.07 [0.04, 0.08 (**0.033**)]
**Covariates**
Age	0.06 [0.05, 0.08 (**2.35** **×** **10**^**−6**^)]	0.06 [0.05, 0.07 (**1.76** **×** **10**^**−6**^)]	0.05 [0.04, 0.07 (**1.01** **×** **10**^**−5**^)]
HHS	1.62 [1.24, 2.00 (**2.09** **×** **10**^**−5**^)]	1.47 [1.10, 1.84 (**8.31** **×** **10**^**−5**^)]	1.52 [1.15, 1.88 (**3.58** **×** **10**^**−5**^)]
mFS	−0.03 [−0.59, 0.53 (0.949)]	0.80 [0.13, 1.13 (0.112)]	0.09 [−0.31, 0.49 (0.883)]
IVH	0.86 [0.51, 1.22 (**0.014**)]	0.80 [0.45, 1.16 (**0.023**)]	0.99 [0.66, 1.33 (**0.003**)]
GCE	0.82 [0.35, 1.28 (0.081)]	0.90 [0.50, 1.39 (**0.043**)]	0.88 [0.44, 1.33 (**0.041**)]
Sex (Male)	−0.24 [−0.58, 0.07 (0.475)]	−0.37 [−0.71, 0.02 (0.282)]	−0.08 [−0.40, 0.24 (0.811)]
Any infection	0.75 [0.42, 1.08 (**0.021**)]	0.85 [0.47, 1.21 (**0.022**)]	0.80 [0.49, 1.11 (**0.011**)]
Aneurysm clipping	0.15 [−0.11, 0.41 (0.567)]	0.25 [−0.004, 0.52 (0.173)]	0.27 [−0.01, 0.55 (0.292)

* *Models based on median cell count for days 1–5*.

# *Data are presented as β coefficient [confidence interval (P-value)]*.

*HHS, Hunt Hess Scale; mFS, modified Fisher Scale; IVH, intraventricular hemorrhage; GCE, global cerebral edema. P-values that are statistically significant are in bold*.

MLR models were also created account for monocytes at day 0 (Supplementary Table 3). Increased day 0 monocyte count was strongly associated with DCI and poor functional outcomes in unadjusted models and after correction for covariates. IVH and infection were associated with DCI. Age, HHS, and infection were associated with worse functional outcomes.

MLR models were created to assess the association between cell count and infection (Table 4). Neutrophil count and NLR were associated with occurrence of infection after correction for covariates. In models adjusted for covariates, monocyte count was not associated with infection. Increased age and higher HHS were associated with infection across all models. Male sex was negatively associated with occurrence of infection in models containing monocytes. When only considering female subjects, monocytes were associated with infection (β = 1.47, 95%CI 0.99, 1.94) with correction for HHS, age, mFS, and IVH. When only considering male subjects, monocyte count was not associated with infection (β =0.19, 95%CI −1.76, 2.12) with correction for HHS, age, mFS, and IVH.

**Table 4:** Associations between leukocytes and presence of infection.

	Neutrophil count^*^	**Monocyte count**	**NLR**
Unadjusted	0.16 [0.12, 0.20 (**4.14** **×** **10**^**−5**^)]^#^	0.44 [0.22, 0.66 (**0.043**)]	0.24 [0.16, 0.32 (**0.002**)]
Adjusted	0.10 [0.06, 0.15 (**0.02**)]	0.05 [−0.21, 0.30 (0.850)]	0.26 [0.17, 034 (**0.005**)]
**Covariates**
Age	0.03 [0.02, 0.04 (**0.007**)]	0.03 [0.03, 0.04 (**0.0002**)]	0.03 [0.02, 0.04 (**0.0005**)]
HHS	0.92 [0.59, 12.6 (**0.006**)]	0.95 [0.63, 1.26 (**0.003**)]	0.87 [0.57, 1.18 (**0.004**)]
mFS	0.10 [−0.38, 0,58 (0.831)]	0.42 [−0.03, 0.88 (0.354)]	0.39 [−0.06, 0.84 (0.381)]
IVH	0.09 [−0.20, 0.39 (0.752)]	0.28 [-0.009, 1.27 (0.302)]	0.24 [−0.03, 0.51 (0.370)]
GCE	−0.57 [−1.42, −0.22 (0.170)]	−0.74 [−1.55, 0.02 (0.065)]	−0.49 [−1.15, 0.35 (0.204)]
Sex (Male)	−0.55 [−0.84, 0.25 (0.066)]	−0.75 [−1.04, −0.47 (**8.63** **×** **10**^**−3**^)]	−0.50 [−1.04, 0.02 (0.064)]

* *Models based on median cell count for days 1–5*.

# *Data are presented as β coefficient [95% confidence interval (P-value)]*.

*HHS, Hunt Hess Scale; mFS, modified Fisher Scale; IVH, intraventricular hemorrhage; GCE, global cerebral edema. P-values that are statistically significant are in bold*.

### Receiver Operating Characteristics (ROC) Curves

**Figure 5:**
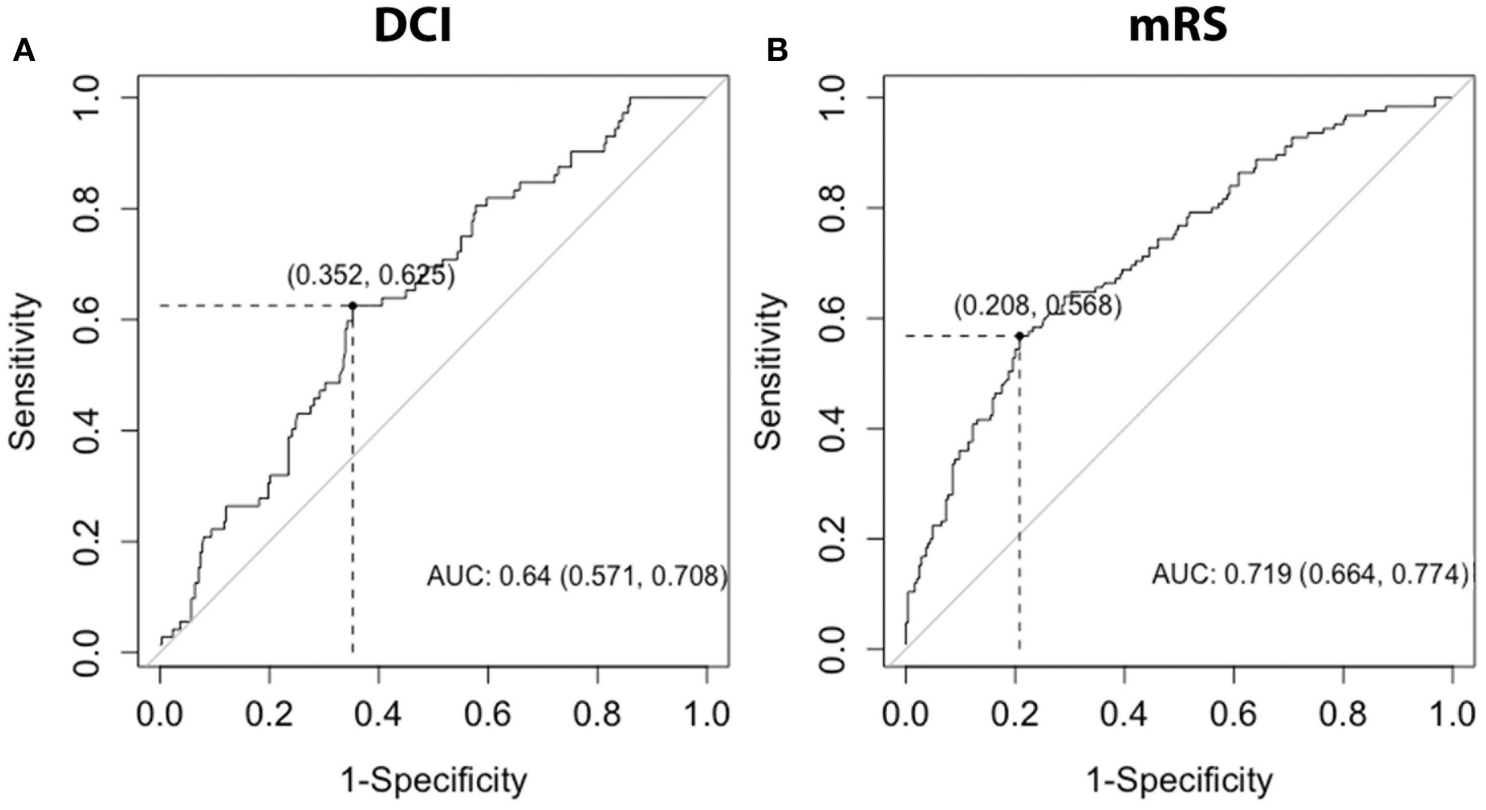
ROC curves for DCI and mRS. ROC curves were generated for the discrimination of DCI or poor discharge mRS, with mRS dichotomized into good (0-3) and poor (4–6) outcomes, using day 0 monocytes. AUC and 95%CI interval were calculated for each ROC curve. DCI, delayed cerebral ischemia; mRS, modified Rankin Scale; AUC, area under the curve.

ROC curves were created considering monocyte count at day 0. For prediction of DCI, monocyte count had an area under the curve (AUC) of 0.640 (Figure 5A). For prediction of poor functional outcomes, monocyte count had an AUC of 0.719 (Figure 5B). Youden index for DCI yielded an optimal cutoff of 0.683 K/mm^3^ (sensitivity 0.625, specificity 0.648). Youden index for mRS yield an optimal cutoff of 0.810 K/mm^3^ (sensitivity 0.568, specificity 0.792). Baseline models were created including HHS, Age, mFS, infection, IVH, gender, and GCE. ROC curves were created for baseline models and baseline models plus day 0 monocyte count. AUC for the baseline model was 0.712 for DCI, which improved to 0.754 when including monocyte count (Supplementary Figure 3A). AUC for the baseline model was 0.858 for outcomes, which improved to 0.877 when including monocyte count (Supplementary Figure 3B).

In females, AUC of the ROC curve for DCI for day 0 monocytes was 0.611 (Supplementary Figure 4A). In males, AUC of the ROC curve for DCI was 0.703 (Supplementary Figure 4B). For females the optimal cutoff for DCI for day 0 monocytes was 0.683 K/mm^3^ (sensitivity 0.571, specificity 0.667). For males, the optimal cutoff for DCI for day 0 monocytes was 0.760 K/mm^3^ (sensitivity 0.760, specificity 0.739). In females, AUC of the ROC curve for mRS was 0.655 (Supplementary Figure 5A). In males, AUC of the ROC curve for mRS was 0.751 (Supplementary Figure 5B). For females, the optimal cutoff for mRS was 0.453 K/mm^3^ (sensitivity 0.891, specificity 0.369). For males, the optimal cutoff for mRS was 0.796 K/mm^3^ (sensitivity 0.595, specificity 0.837). These results are summarized in Supplementary Table 4.

## Discussion

This study demonstrates that peripheral leukocytes robustly distinguish outcomes after SAH, with persistently elevated WBCs, neutrophils, and NLR in those with DCI and poor outcomes. Although leukocytes were affected by clinical severity, there was an independent effect of cell counts on outcomes. Monocyte count (both on admission and medians from day 1–5) robustly predicted DCI and outcomes, in a sex dependent fashion. We found an association between infection and outcome and importantly demonstrated a link between neutrophils and NLR with the occurrence of infection after SAH.

Monocytes remained significantly elevated across all study days in subjects with DCI and poor functional outcomes. Peripheral monocyte count has been shown to increase early after brain injury (33). Monocytes play a beneficial role by removing debris in the subarachnoid space (34), however increased monocyte counts have also been associated with worse outcome after ICH (18). The pathophysiology underlying this association is unclear. However, monocytes have been linked to hematoma expansion in patients with ICH (35). Monocytes are coated with physiological anticoagulants such as thrombomodulin and tissue factor pathway inhibitor 2 (36, 37). A mouse model also found decreased extent of bleeding in an ICH model with impaired CCL2-CCR2 chemoattractant system (38). Peripheral monocytes play an important role in cerebral inflammation after injury in stroke, ICH, Alzheimer's and status epilepticus (34, 35, 39–42). Mice deficient in Toll-like receptor 4 (TLR4) demonstrated decreased recruitment of monocytes after ICH associated with decreased perihematomal edema (39). Therefore, monocytes may play a role both in hemorrhage expansion and inflammation after SAH. Indeed, the innate immune system has been shown to be activated early after SAH, with cells trafficking to the brain from the periphery (43). CD14^+^CD16^−^ monocytes (22) as well as monocytes expressing programmed death-1 (PD-1) are thought to play a role in the development of vasospasm, as blocking their ingress to the CNS after SAH attenuated vasospasm (44).

The early (day 0 and 1) increase among subjects with DCI and poor functional outcomes distinguished monocytes and NLR. Indeed, increased monocytes were robustly associated with DCI and poor functional outcome both considering median cell counts over days 1–5 (Tables 2, 3) and day 0 (Supplementary Table 3). The ability of monocytes to predict delayed cerebral infraction and poor outcomes after SAH has recently been demonstrated in a cohort of 204 patients (27). These authors found an optimal cutoff of 0.60 to discriminate development of cerebral infarction and poor outcomes after SAH, with AUC of 0.622. We found an improved AUCs for discrimination of DCI (0.640) and poor functional outcome (0.719). Our results also suggest a higher monocyte count may improve discrimination of DCI (0.683) and functional outcomes (0.810). When adding day 0 monocyte count to a baseline model including HHS, age, mFS, infection, IVH, and GCE, the AUCs further improved to 0.754 to DCI and 0.877 for functional outcome (Supplementary Figure 3).

Monocyte counts were significantly higher in males (Figure 4). Of note, monocytes had a better ability to discriminate DCI (AUC 0.703 vs. 0.611) and outcomes (AUC 751 vs. 655) in males compared with females. Monocyte function is sex specific and absolute numbers of monocytes have been shown to be higher in males than females (45). Several studies have also reported more robust cytokine production in male compared with female monocytes (46–48). Sex hormones impact immune cell function. Estrogen has been found to promote M2 polarization, which was dependent on the function of estrogen receptor α (49). We theorize that male monocytes are more likely to undergo M1 polarization with increased pro-inflammatory cytokine production after SAH. Furthermore, unlike neutrophils and NLR, monocyte count was not significantly associated with occurrence of infection (Table 4). Interestingly, an association between infection and monocyte count was only found in women and not seen in males. To summarize, monocytes increased steadily in both sexes. Monocyte counts were predictive of the occurrence of DCI in men. Monocyte counts were predictive of the occurrence of infections in women. This suggests that there are sex-specific effects of monocytes after SAH. Further examination of the underlying pathophysiology of these differences should be pursued.

An elevated NLR likely represents dysregulation of the immune system reflecting an increased innate and attenuated adaptive immune response (8, 50). In addition to playing a role in ICH and stroke (26, 51) an elevated NLR has been linked to outcomes in patients with cancer, myocardial infarction, and sepsis (31, 52–56). Neutrophils infiltrate the cerebral vasculature within 10 min after SAH, and reduction of neutrophil levels or activity improves vascular integrity and outcomes in animal models (14, 57). In addition to producing pro-inflammatory cytokines, neutrophils generate substantial oxidative stress by releasing factors such as myeloperoxidase, which can generate hypochlorous acid and consume nitric oxide resulting in impaired vasodilation (58). Isoprostanes generated by neutrophil mediated oxidative stress can also result in vasoconstriction (59, 60). Indeed, neutrophils may also contribute to early cerebral hypoperfusion after SAH (61).

This study builds upon results from prior studies, which have demonstrated the role of peripheral leukocytes after SAH. While several important studies have shown that WBC count has an association with DCI (62, 63), herein we have provided a comprehensive assessment of the role of differential cell counts on DCI and functional outcomes. Our assessments of the role of sex differences and associations with infection have also not been addressed in prior studies. We also support findings from previous studies indicating an association between elevated NLR and DCI (23, 25) and outcomes (24, 26). Our study has key differences with these reports, with a main difference being that most studies have exclusively assessed the role of early leukocyte counts. The largest study to date, which evaluated 1067 SAH patients, found an admission NLR cutoff of 5.9 to be highly predictive of the development of DCI with no association between NLR and functional outcomes (25). Our results demonstrate that WBCs, neutrophils, and NLR remain elevated in patients with DCI and poor functional outcome for a week after the initial bleed and these differences increase after the first two post-bleed days (Figure 1). This is in line with granulocytes being persistently elevated in the brain for at least a week after SAH in a rat model (64). We did not find associations with outcomes using early (day 0) WBCs, neutrophils, and NLR but found robust associations using median values over 5 days (Tables 2, 3). We suggest that the earliest time point for WBCs and NLR may not be as tightly linked to ultimate clinical outcome.

There was an association between neutrophils and NLR and the occurrence of infection after SAH, while lymphocytes were associated with fewer infections (Table 4). Elevated NLR plays a role in post SAH immunosuppression (26) and contributes to the risk of pneumonia (65). Neutrophils may play a role in the suppression of the adaptive immune system (50). The immunosuppressive capacity of some neutrophils is due to the ability to inhibit T-cell activation, proliferation, and effector functions (66). However, it is unclear from our results whether neutrophilia precedes infection, or whether early infections drive persistently elevated neutrophils and NLR. The role of lymphocytes in the injured brain is complex and incompletely understood. T cells infiltrate 48–96 h after injury and peak around 5 days (67, 68) in mice but have been found in the CSF after ICH in humans after 6 h (69). T cells comprise a heterogenous population and have been shown to have both protective and deleterious roles (70). Both pro-inflammatory (γδT cells) and immunosuppressive regular T cells (Tregs) traffic to the area of hemorrhage (32). γδT cells have been shown to contribute to injury after ischemic stroke mainly due to the production of IL-17 (71). Tregs may provide benefits against the late tissue damage (72), while in the earlier time frame they have been shown to modulate the activity of invading pro-inflammatory cells (73). The role of γδT cells may be primarily in the periphery rather than brain parenchyma as they accumulate in the leptomeninges and control trafficking of other inflammatory cells (74). While our study is not able to distinguish between lymphocyte subtypes, it is possible that the serum lymphocytes and decreased NLR associated with good outcomes and decreased risk of infection outcomes are primarily Tregs. We suspect that increased neutrophils and decreased lymphocytes are key factor leading to risk of infection after SAH, with this serving as a major determinant of clinical outcomes.

A major caveat to the interpretation of the results presented herein is the prevalent use of corticosteroids. At our institution, patients with aneurysmal SAH are typically treated with a standard regimen of corticosteroids during the first week of hospitalization, affecting most subjects in this study. Corticosteroids have an important effect on circulating leukocytes. Corticosteroid treatment increases neutrophils due to increased entrance from bone marrow and decreased vascular removal. However, monocytes, lymphocytes, basophils, and eosinophils are decreased after steroid treatment. This effect is thought to be due to redistribution of these cells, although lymphocytes are known to undergo steroid induced apoptosis (75). In addition, corticosteroids have potent anti-inflammatory effects. Corticosteroids inhibit recruitment of neutrophils and monocytes (76) and inhibit neutrophil adherence to capillary endothelial cells by decreasing expression of adhesion molecules and the synthesis and release of prostaglandins (77, 78). It is therefore very likely that corticosteroid treatment has affected the cell counts used in this study. As nearly all patient received corticosteroid treatment, it is not possible to control for this effect in our multivariate regression models. Additional studies will be needed to confirm the findings described herein in SAH subjects not treated with corticosteroids.

In addition to the effect of corticosteroids, this study has several other important limitations. This is a single center study, which limits its generalizability. This study is also retrospective, and it is therefore prone to selection bias. Given that multiple comparisons among cell types and across different days were made, there is a risk of type I error. We have adjusted *P-*values considered to be significant in univariate analyses for cell counts for multiple comparisons using a Bonferroni correction to mitigate this risk. We attempt to explore the pathophysiological basis for the connection between leukocytes and outcomes, however our study is limited to clinically available differentials. Subtypes of each leukocyte may play distinct roles (e.g., lymphocyte subtypes may be beneficial or harmful). Future flow cytometry studies will be used to answer questions about specific cell types. Several variables of interest in the study require qualitative assessment such as GCE, mFS, and DCI. We attempt to control for any variability and subjectivity by having all variables adjudicated by multiple reviewers.

This study determined the time course of peripheral leukocyte responses after SAH. We found that monocytes were associated with DCI and poor functional outcome, in a sex dependent fashion. Monocytes were elevated in men compared to women. In men, monocyte counts predicted the occurrence of DCI, while in women monocyte counts were associated with infection. Both neutrophil count and NLR were associated with worse outcomes. We suspect increased NLR (reflective of increased neutrophils and decreased lymphocytes) results in an immunosuppressive environment that contributes to infection risk, and this plays a role in outcomes. We suggest that monocytes (especially in males) play a key role in driving systemic and central inflammation after SAH, while neutrophils and NLR affect outcomes by modulating infection risk. Monocyte count may help to predict DCI and outcomes after DCI and may serve as a target for therapeutic intervention.

## Data Availability Statement

The data supporting the conclusions of this article will be made available by the authors upon reasonable request.

## Ethics Statement

The studies involving human participants were reviewed and approved by University of Texas McGovern School of Medicine Institutional Review Board. The patients/participants provided their written informed consent to participate in this study.

## Author Contributions

AG designed the study, acquired and analyzed data, and drafted the article. JS analyzed data and helped to draft the article. ES acquired and analyzed data and helped to draft the article. AP acquired data and revised the article. AA acquired data and revised the article. S-BK contributed to study design and revised the article. LM contributed to study design and revised the article. HC contributed to study designed, analyzed data, and critically revised the article. All authors contributed to the article and approved the submitted version.

## Conflict of Interest

The authors declare that the research was conducted in the absence of any commercial or financial relationships that could be construed as a potential conflict of interest.

## Publisher's Note

All claims expressed in this article are solely those of the authors and do not necessarily represent those of their affiliated organizations, or those of the publisher, the editors and the reviewers. Any product that may be evaluated in this article, or claim that may be made by its manufacturer, is not guaranteed or endorsed by the publisher.
