# Supplementary material for: Time Course of Peripheral Leukocytosis and Clinical Outcomes After Aneurysmal Subarachnoid Hemorrhage
Source: Front Neurol. 2021 Jul 26;12:694996. doi: 10.3389/fneur.2021.694996 (PMC8350167; doi:10.3389/fneur.2021.694996)
Supplement: Supplementary file 1 [file Data_Sheet_1.pdf]

Supplementary Material

I. Supplementary Figure 1
II. Supplementary Figure 2
III. Supplementary Figure 3
IV. Supplementary Figure 4
V. Supplementary Figure 5
VI. Supplementary Table 1
VII. Supplementary Table 2
VIII. Supplementary Table 3
IX. Supplementary Table 4

Supplementary Figure 1

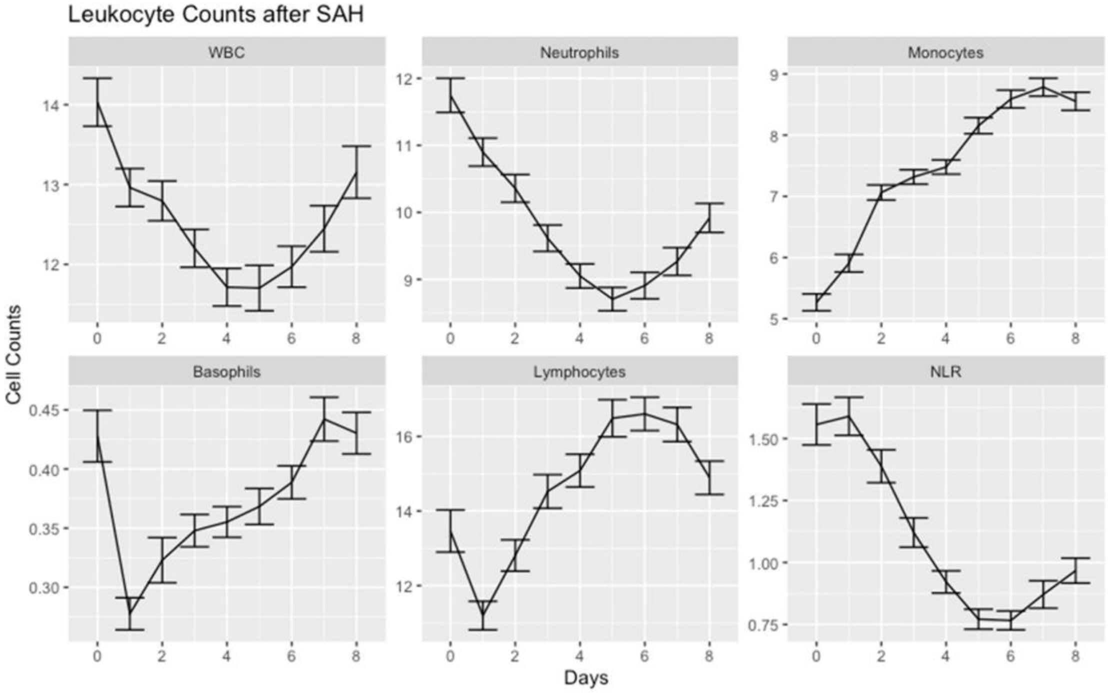

Supplementary Figure 1. Temporal profile of leukocytes after SAH. Leukocytes counts (mean and standard deviation) are shown from day of SAH (day 0) through day 8. Cells are in units of 1,000 per mm^3. **P*<0.05, ***P*<0.01, †*P*<0.001. Abbreviations: subarachnoid hemorrhage (SAH), white blood cells (WBC), neutrophil-to-lymphocyte ratio (NLR).

Supplementary Figure 2

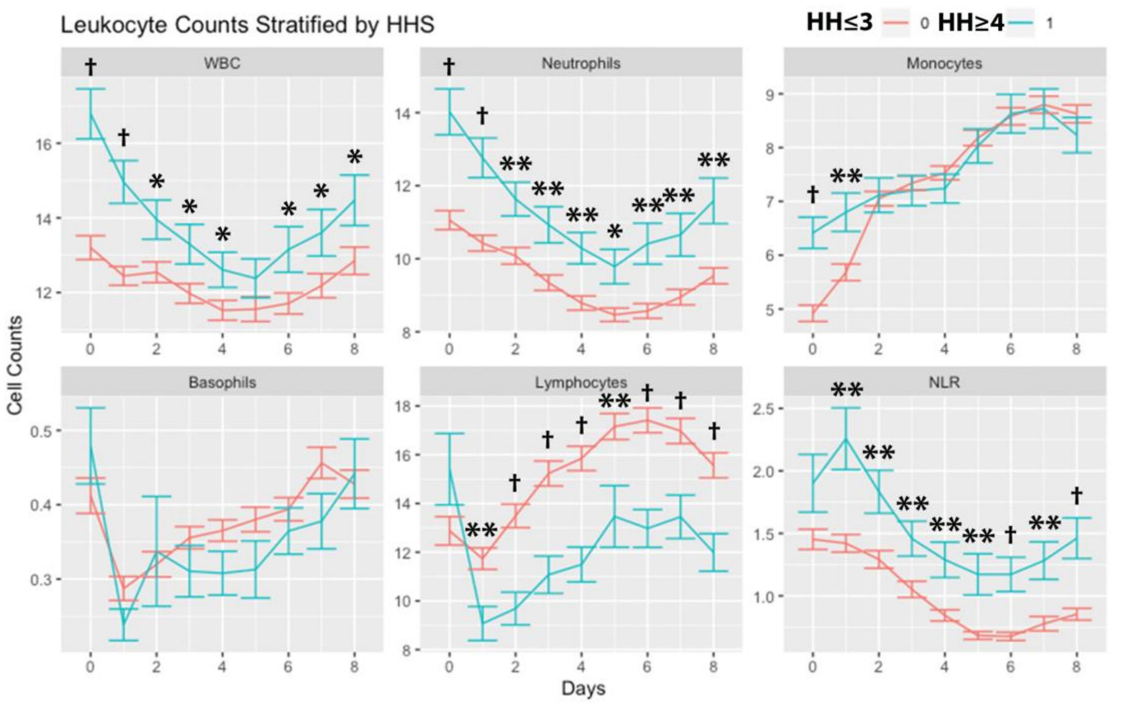

Supplementary Figure 2. Leukocyte counts stratified by Hunt Hess Scale (HHS). Leukocytes counts (mean and standard deviation) are shown from day of SAH (day 0) through day 8. HHS is dichotomized to low grade (≤3) and high grade (4-5). Cells are in units of 1,000 per mm^3. *P<0.05, **P<0.01, †P<0.001. Abbreviations: Hunt Hess scale (HHS), subarachnoid hemorrhage (SAH), white blood cells (WBC), neutrophil-to-lymphocyte ratio (NLR).

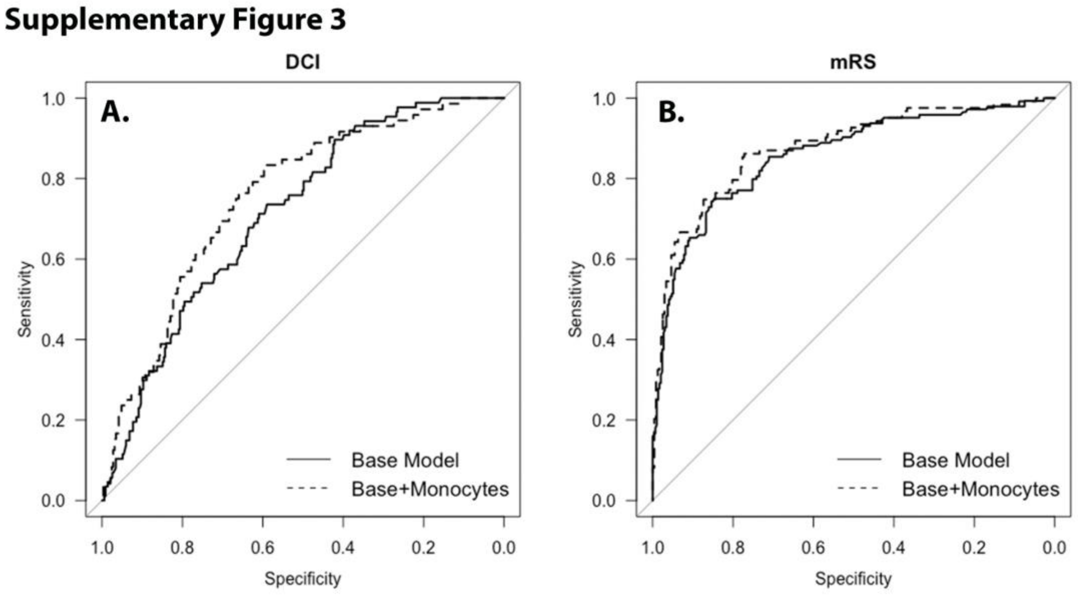

Supplementary Figure 3. ROC for DCI and mRS. ROC curves were generated for discrimination of DCI or discharge mRS, with mRS dichotomized into good (0-3) and poor (4-6) outcomes, using day 0 monocytes. AUC and 95%CI were calculated for each ROC curve. Baseline model included HHS, age, mFS, infection, IVH, GCE, and gender. Day 0 monocyte counts were added to each model. Abbreviations: delayed cerebral ischemia (DCI), modified Rankin Scale (mRS), area under the curve (AUC), Hunt Hess Scale (HHS), modified Fisher Scale (mFS), intraventricular hemorrhage (IVH), global cerebral edema (GCE).

Supplementary Figure 4

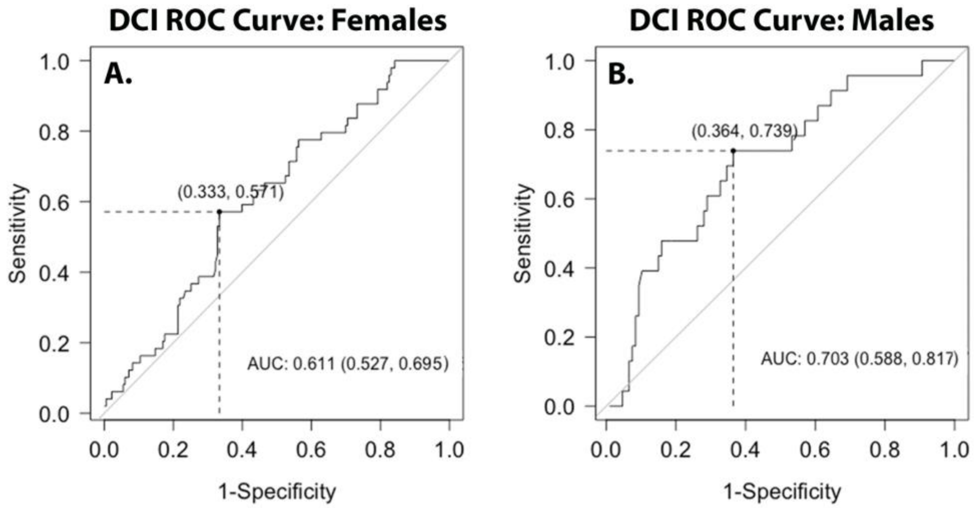

Supplementary Figure 4. ROC curves for DCI according to sex. ROC curves were generated for the discrimination of DCI using day 0 monocytes. Subjects were divided into females (A) and males (B). AUC and 95%CI interval were calculated for each ROC curve. Abbreviations: delayed cerebral ischemia (DCI), area under the curve (AUC).

Supplementary Figure 5

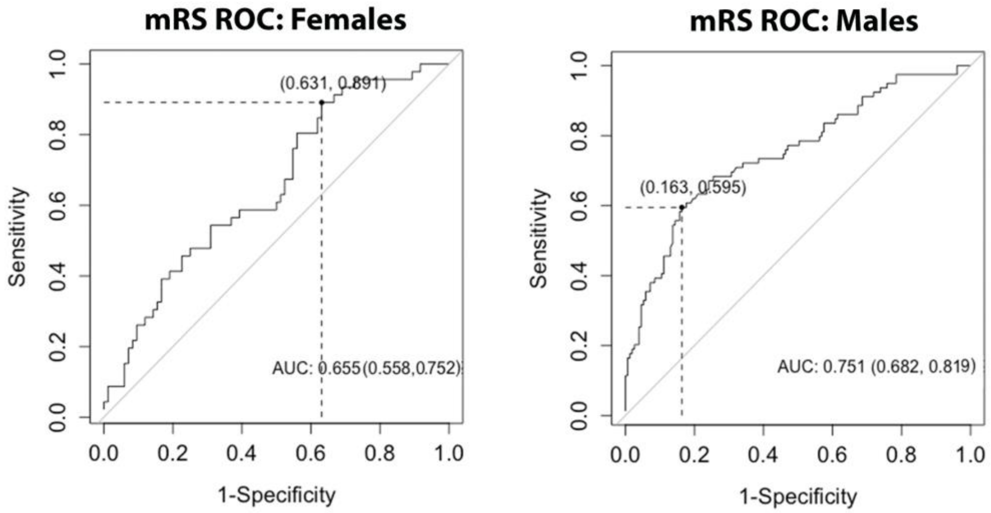

Supplementary Figure 5. ROC curves for mRS according to sex. ROC curves were generated for the discrimination of mRS (dichotomized as good (≤3) and poor (4-6)) using day 0 monocytes. Subjects were divided into females (A) and males (B). AUC and 95%CI interval were calculated for each ROC curve. Abbreviations: modified Rankin Scale (mRS), area under the curve (AUC).

Supplementary Table 1: Univariate models for DCI assessing each cell type across all days.

	WBC	Neutrophils	Monocytes	Basophils	Lymphocytes	NLR
Day 0	0.079	0.097	**$7.03x10^{-4}$**	0.181	0.683	0.150
Day 1	0.038	0.014	0.024	0.178	0.880	0.643
Day 2	$1.23x10^{-3}$	**$4.77x10^{-5}$**	**$7.64x10^{-4}$**	0.215	0.685	0.060
Day 3	**$1.35x10^{-4}$**	**$2.24x10^{-6}$**	**$1.58x10^{-4}$**	0.685	0.753	0.063
Day 4	**$1.20x10^{-4}$**	**$7.47x10^{-7}$**	**$4.75x10^{-6}$**	0.549	0.580	0.043
Day 5	0.017	**$1.64x10^{-4}$**	**$4.64x10^{-5}$**	0.943	0.800	0.079
Day 6	0.015	$1.54x10^{-3}$	**$7.69x10^{-4}$**	0.908	0.929	0.319
Day 7	0.019	$5.20x10^{-4}$	**$5.28x10^{-4}$**	0.153	0.592	0.070
Day 8	$1.91x10^{-3}$	$7.37x10^{-7}$	**$2.79x10^{-4}$**	0.470	0.721	0.212

P-values are depicted for each univariate model. Given that multiple comparisons (54) are being made, a *P*-values of $< 9.3x10^{-4}$ are considered to be significant. Values highlighted in red indicate a significant association with DCI.

Supplementary Table 2: Univariate models for modified Rankin Scale (mRS) outcomes assessing each cell type across all days.

	WBC*	Neutrophils	Monocytes	Basophils	Lymphocytes	NLR
Day 0	**$2.08x10^{-6}$**	**$8.43x10^{-5}$**	**$9.47x10^{-14}$**	$8.17x10^{-3}$	$3.18x10^{-3}$	0.153
Day 1	**$4.97x10^{-5}$**	**$2.36x10^{-4}$**	**$2.71x10^{-4}$**	0.256	0.200	0.031
Day 2	**$2.84x10^{-6}$**	**$6.68x10^{-7}$**	**$1.05x10^{-4}$**	0.178	0.578	**$2.30x10^{-5}$**
Day 3	**$4.52x10^{-8}$**	**$4.03x10^{-9}$**	**$3.98x10^{-4}$**	0.287	0.469	**$1.29x10^{-4}$**
Day 4	**$7.43x10^{-9}$**	**$2.21x10^{-11}$**	**$3.72x10^{-7}$**	0.042	0.606	**$7.55x10^{-7}$**
Day 5	**$4.37x10^{-6}$**	**$3.21x10^{-10}$**	**$3.93x10^{-4}$**	$5.69x10^{-3}$	0.342	**$5.99x10^{-7}$**
Day 6	**$3.08x10^{-8}$**	**$4.72x10^{-10}$**	**$3.33x10^{-6}$**	0.046	0.330	**$1.21x10^{-7}$**
Day 7	**$1.46x10^{-8}$**	**$4.61x10^{-11}$**	**$2.67x10^{-5}$**	0.026	0.337	**$2.52x10^{-6}$**
Day 8	**$7.08x10^{-8}$**	**$8.60x10^{-10}$**	**$1.30x10^{-4}$**	0.350	0.140	$5.86x10^{-3}$

Outcomes are dichotomized as good (mRS 0-3) and bad (mRS 4-6). *P*-values are depicted for each univariate model. Given that multiple comparisons (54) are being made, a *P*-values of $< 9.3x10^{-4}$ are considered to be significant. Values highlighted in red indicate a positive association with poor mRS (4-6).

Supplementary Table 3. Associations between day 0 monocyte counts and outcomes

	DCI*	mRS
Unadjusted	0.75 [0.52, 0.98 (**0.001**)][#]	1.80 [1.52, 2.08 (**1.93×10^{-10}**)
Adjusted	0.80 [0.51, 1.09 (**0.006**)]	1.21 [0.86, 1.56 (**5.49×10^{-4}**)
Covariates		
Age	-0.01 [-0.023, 0.0008 (0.283)]	0.07 [0.06, 0.09 (**1.42×10^{-8}**)]
HHS	-0.78 [-1.16, 0.40 (0.059)]	2.20 [1.83, 2.58 (**4.50×10^{-9}**)]
mFS	0.41 [-0.02, 0.84 (0.339)]	0.29 [-0.11, 0.70 (0.468)]
IVH	1.15 [0.79, 1.51 (**0.001**)]	0.53 [0.20, 0.87 (0.113)]
GCE	0.56 [0.05, 0.96 (0.163)]	0.65 [0.18, 1.13 (0.172)]
Sex (Male)	-0.07 [0.38, 0.24 (0.824)]	0.32 [-0.007, 0.64 (0.328)]
Any Infection	0.98 [0.66, 1.29 (**0.002**)]	0.75 [0.42, 1.09 (**0.03**)]

*Models based on monocyte count at day 0. [#]Data are presented as β coefficient [95% confidence interval (*P* value)]. Abbreviations: HHS (Hunt Hess Scale), mFS (modified Fisher Scale), IVH (intraventricular hemorrhage), GCE (global cerebral edema).

Supplementary Table 4. ROC analysis and Youden index cutoff values.

	Day 0 Monocyte Counts			
		All Patients	Females	Males
DCI	AUC	0.640 (0.571, 0.708)	0.611 (0.527, 0.695)	0.703 (0.588, 0.817)
	Cutoff	0.683 (se 0.625, sp 0.648)	0.683 (se 0.571, sp 0.667)	0.760 (se 0.760, sp 0.739)
mRS	AUC	0.719 (0.664, 0.774)	0.655 (0.558, 0.752)	0.751 (0.682, 0.819)
	Cutoff	0.810 (se 0.568, sp 0.792)	0.453 (se 0.891, sp 0.369)	0.796 (se 0.595, sp 0.837)

Abbreviations: delayed cerebral ischemia (DCI), modified Rankin Scale (mRS), area under the curve (AUC), sensitivity (se), specificity (sp).
